# The B domain of protein A retains residual structures in 6 M guanidinium chloride as revealed by hydrogen/deuterium‐exchange NMR spectroscopy

**DOI:** 10.1002/pro.4569

**Published:** 2023-02-14

**Authors:** Saeko Yanaka, Maho Yagi‐Utsumi, Koichi Kato, Kunihiro Kuwajima

**Affiliations:** ^1^ Exploratory Research Center on Life and Living Systems (ExCELLS) and Institute for Molecular Science (IMS), National Institutes of Natural Sciences, Myodaiji Okazaki Aichi Japan; ^2^ Department of Functional Molecular Science School of Physical Sciences, SOKENDAI (the Graduate University for Advanced Studies), Myodaiji Okazaki Aichi Japan; ^3^ Graduate School of Pharmaceutical Sciences, Nagoya City University, Mizuho‐ku Nagoya Aichi Japan; ^4^ Department of Physics School of Science, University of Tokyo Bunkyo‐ku Tokyo Japan; ^5^ Present address: Graduate School of Pharmaceutical Sciences Kyushu University Fukuoka Japan

**Keywords:** 2D NMR, hydrogen/deuterium exchange, protein folding, residual structure, unfolded state

## Abstract

The characterization of residual structures persistent in unfolded proteins is an important issue in studies of protein folding, because the residual structures present, if any, may form a folding initiation site and guide the subsequent folding reactions. Here, we studied the residual structures of the isolated B domain (BDPA) of staphylococcal protein A in 6 M guanidinium chloride. BDPA is a small three‐helix‐bundle protein, and until recently its folding/unfolding reaction has been treated as a simple two‐state process between the native and the fully unfolded states. We employed a dimethylsulfoxide (DMSO)‐quenched hydrogen/deuterium (H/D)‐exchange 2D NMR techniques with the use of spin desalting columns, which allowed us to investigate the H/D‐exchange behavior of individually identified peptide amide (NH) protons. We obtained H/D‐exchange protection factors of the 21 NH protons that form an α‐helical hydrogen bond in the native structure, and the majority of these NH protons were significantly protected with a protection factor of 2.0–5.2 in 6 M guanidinium chloride, strongly suggesting that these weakly protected NH protons form much stronger hydrogen bonds under native folding conditions. The results can be used to deduce the structure of an early folding intermediate, when such an intermediate is shown by other methods. Among three native helical regions, the third helix in the C‐terminal side was highly protected and stabilized by side‐chain salt bridges, probably acting as the folding initiation site of BDPA. The present results are discussed in relation to previous experimental and computational findings on the folding mechanisms of BDPA.

## 1 INTRODUCTION

The problem of protein folding is a fundamental issue in protein science. Although highly accurate predictions of three‐dimensional protein structures have recently been realized by neural network‐based methods (Jumper et al. 2021; Baek et al. 2021), such machine‐learning approaches cannot provide fundamental knowledge about the molecular mechanisms of protein folding. Therefore, it is still important to understand the protein folding processes by means of experimental and theoretical studies. Early theoretical studies assumed that the protein folding starts from a completely unfolded randomly coiled state as exemplified by Levinthal's gedankenexperiment (Levinthal 1969). The experimental approaches to protein denaturation and folding, including Anfinsen's historical experiments (Anfinsen 1973), used a high concentration of a strong denaturant, such as 8 M urea or 6 M guanidinium chloride (GdmCl), as a condition to fully unfold proteins, and hence, the proteins were assumed to be in the fully unfolded random coil, in which there was no trace of the native regular conformations (Tanford 1968, 1970). However, more recent experimental studies have provided several lines of evidence for the persistence of residual structures in the fully unfolded (U) state in concentrated urea or GdmCl for different proteins, raising the possibility that those residual structures may reflect the initial starting state in protein folding (Neri et al. 1992; Schwalbe et al. 1997; Klein‐Seetharaman et al. 2002; Wirmer et al. 2006; Dar et al. 2011). Therefore, detailed characterization of the residual structures in the U state is indispensable for better understanding of the molecular mechanisms of protein folding, but it is extremely difficult to characterize the residual structures in detail, because of the lack of appropriate experimental methods.

Recently, we developed a new dimethylsulfoxide (DMSO)‐quenched hydrogen/deuterium (H/D)‐exchange two‐dimensional (2D)‐NMR method to detect and characterize the residual structures of a protein retained in the U state in concentrated denaturant (Chandak et al. 2013; Kuwajima et al. 2022). In this method, spin desalting columns are used to quickly exchange the medium (e.g., 6 M GdmCl) for a DMSO solution, which quenches the H/D‐exchange reactions (Zhang et al. 1995), and then the 2D NMR spectra of the protein are measured in the DMSO solution. We previously used this method to investigate the H/D‐exchange kinetics of individually identified peptide amide (NH) protons of ubiquitin (Ub), a small α/β protein, dissolved in 6 M GdmCl, after carrying out the backbone resonance assignment of the 2D NMR spectrum of the protein in DMSO solution in advance (Yagi‐Utsumi et al. 2020). Our results demonstrated that Ub retains residual hydrogen‐bonded (H‐bonded) structures even in 6 M GdmCl, casting doubt on the traditional theory of protein folding. We have thus concluded that the native‐like residual structures protect certain NH groups in the central helix and the N‐terminal hairpin in unfolded Ub and that they may play an important role at an initial stage of kinetic refolding from the U state of the protein (Yagi‐Utsumi et al. 2020).

In this study, we extend the application of this DMSO‐quenched H/D‐exchange method to a protein with a different structural class to examine the general existence of such residual structures in the U state in 6 M GdmCl. We chose the B domain of protein A (BDPA) from *Staphylococcus aureus*, which is the third of five tandem immunoglobulin‐binding domains (Boyle 1990), each of which adopts a homologous three‐helix bundle structure (Gouda et al. 1992). BDPA is one of the most extensively studied all‐α proteins; BDPA has been structurally characterized not only as an immunoglobulin‐binding protein (Kato et al. 1993; Deisenhofer 1981; Gouda et al. 1998) but also from the perspective of protein folding and stability. The earlier studies of the BDPA folding were carried out on the basis of a simple two‐state model between the native (N) and the U states without any stable folding intermediate (Sato et al. 2004, 2006; Myers and Oas 2001; Arora et al. 2004; Huang et al. 2007, 2009; Bottomley et al. 1994; Bai et al. 1997; Cedergren et al. 1993; Dimitriadis et al. 2004). However, recent experimental and computational studies have demonstrated the presence of an early α‐helical folding intermediate during the kinetic refolding of the protein, and have indicated distinct kinetic and thermodynamic stabilities among the three helices (Vu et al. 2004; Davis et al. 2015; Garcia and Onuchic 2003; Lei et al. 2008; Shao and Gao 2011; Shao and Zhu 2017; Jani et al. 2016; Shao 2014). In addition, analyses using fluorescence spectroscopic techniques have revealed the structural heterogeneity of unfolded BDPA in concentrated GdmCl, and have implied the presence of residual structures in the U state (Otosu et al. 2017; Oikawa et al. 2013, 2015). However, these studies did not provide structural information at the amino acid residue level. Therefore, we here applied our newly developed DMSO‐quenched H/D‐exchange method to investigate the presence of H‐bonded residual structures in unfolded BDPA in 6 M GdmCl by measuring the degrees of protection conferred to individual NH groups against the H/D exchange with a solvent deuteron. We show that native‐like H‐bonded structures are partially retained in unfolded BDPA in 6 M GdmCl, especially in the third α‐helix, and that these residual structures are incipient, fluctuating and mostly one‐turn helices, which exhibit no significant α‐helical circular dichroism (CD) bands. We discuss the present results in comparison with the previous experimental and computational studies on the molecular mechanisms of the BDPA folding.

## 2 RESULTS

### 2.1 The amino acid sequence and the equilibrium unfolding of B domain of protein A

**FIGURE 1:**
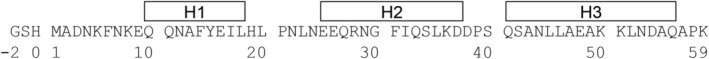
The amino acid sequence of BDPA. Rectangles, H1, H2, and H3 represent the three α‐helical regions in native BDPA (PDB code: 1BDD).

The amino acid sequence of BDPA used in the present study is shown in Figure 1. To ensure that the amino acid residue numbering of BDPA is consistent with the previous studies(Gouda et al. 1992; Sato et al. 2004), we take the fourth residue (Met) from the N‐terminus as position 1. The three α‐helices are designated as H1, H2, and H3, which correspond to Gln10‐Leu18, Glu25‐Asp37, and Gln41‐Gln56, respectively.

**FIGURE 2:**
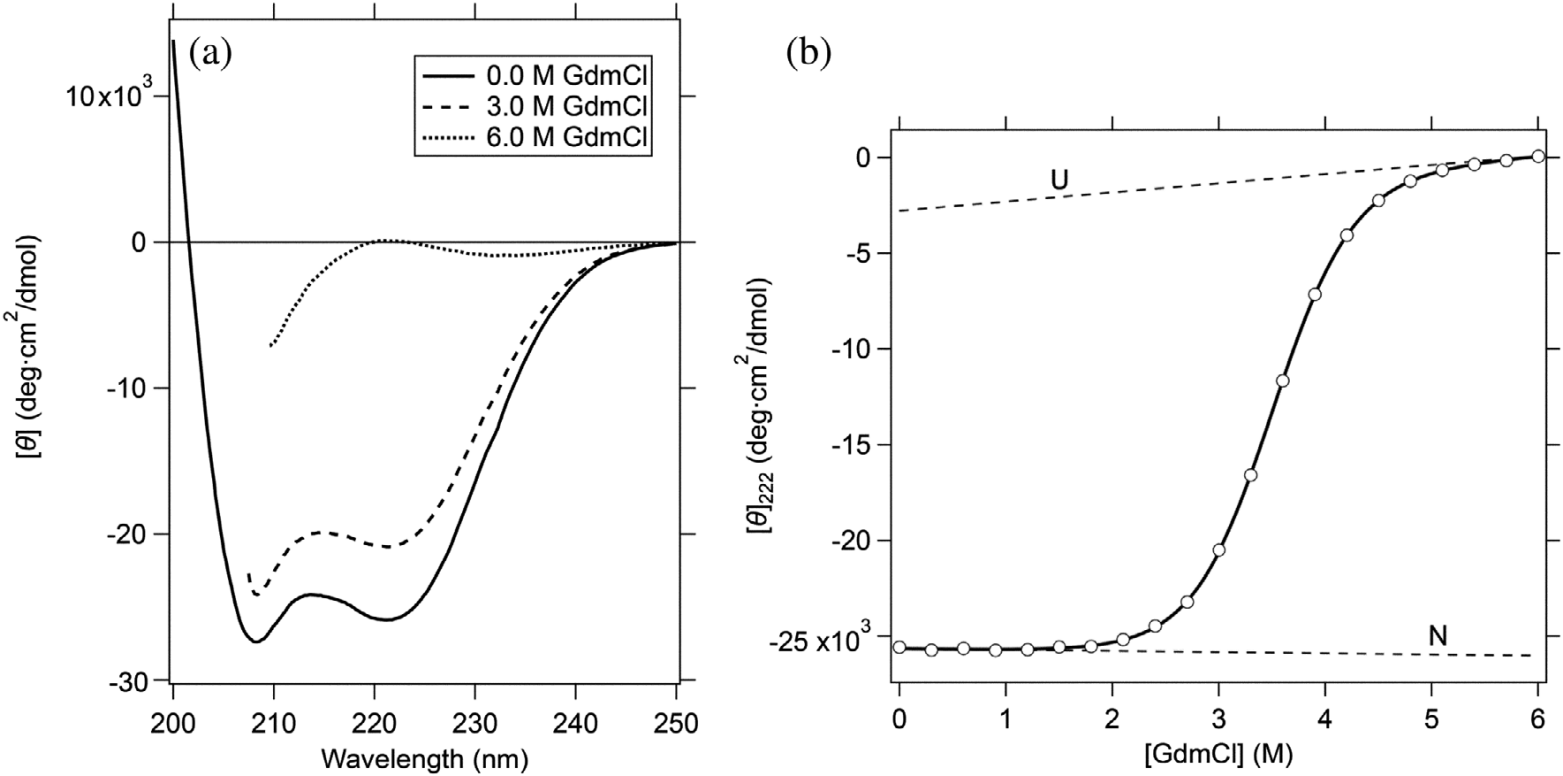
The CD spectra of BDPA at 0.0, 3.0, and 6.0 M GdmCl (a), and the unfolding transition curve measured by the mean residue ellipticity at 222 nm (0.10 M NaCl–20 mM HCOONa in 90% D_2_O/10% H_2_O at pH* 3.4 and 15.0°C) (b). The solid line in (b) is the theoretical curve best fitted to Equation (6), and two broken lines represent the ellipticity values in the pure N and the pure U states.

Figure 2 shows CD spectra of BDPA in the peptide region at three different concentrations (0.0, 3.0, and 6.0 M) of GdmCl (Figure 2a), and a GdmCl‐induced unfolding transition curve of the protein measured by the mean residue ellipticity at 222 nm (Figure 2b). BDPA was fully unfolded at 6.0 M GdmCl in 90% D_2_O/10% H_2_O at pH* 3.4 and 15°C, the condition employed for the peptide NH H/D‐exchange experiments in the present study; pH* indicates the pH meter reading. The equilibrium unfolding parameters, the Gibbs free energy change of unfolding at 0 M GdmCl, Δ*G*°_NU_, and the cooperativity parameter of unfolding, *m*_NU_, evaluated from Figure 2b using nonlinear least‐squares analysis with Equation (6) are listed in Table 1. These equilibrium unfolding parameters show reasonable agreement with the corresponding parameter values in previous studies (Table 1) (Myers and Oas 2001; Arora et al. 2004).

**TABLE 1:** Equilibrium unfolding parameters of BDPA

Δ*G*°_NU_ (kcal/mol)	*m*_NU_ (kcal/mol/M)	Solution conditions	Reference
5.30 ± 0.04	1.52 ± 0.02	0.10 M NaCl in 90% D_2_O/10% H_2_O at pH* 3.4 and 15°C	This study
4.3 ± 0.2	1.5 ± 0.1	0.10 M NaCl in D_2_O at pH* 5.0 and 37°C	Myers and Oas (2001)

### 2.2 NMR spectral assignment of B domain of protein A in the dimethylsulfoxide solution

**FIGURE 3:**
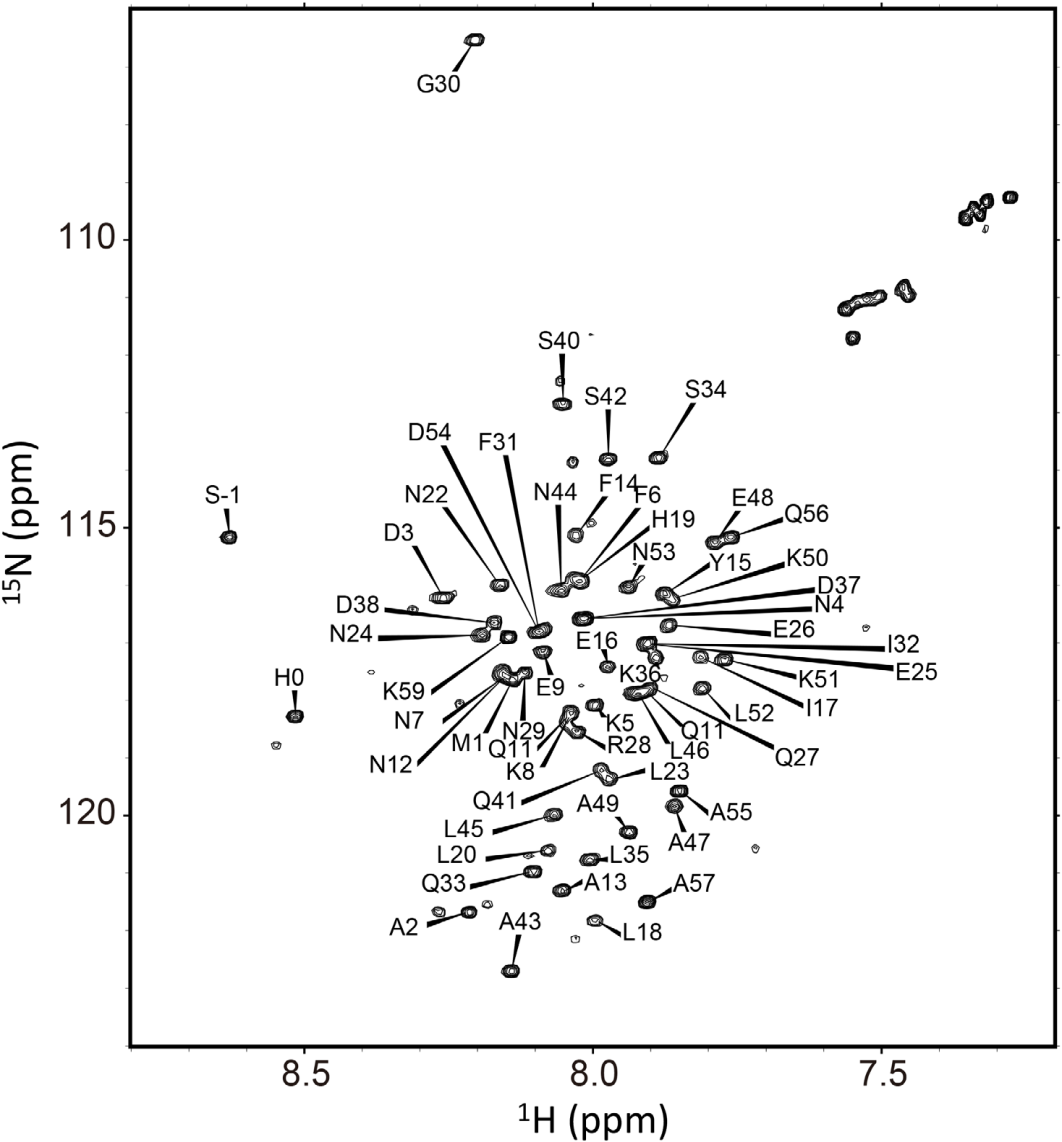
The ^1^H–^15^ N HSQC spectra of uniformly ^13^C/^15^ N‐labeled BDPA in the DMSO/H_2_O solution (94.5% (v/v) DMSO‐*d*_6_/5% (v/v) H_2_O/0.5% (v/v) DCA‐*d*_2_).

We used the DMSO‐quenched method in the present H/D‐exchange experiments, in which the H/D‐exchange reaction of ^15^ N‐labeled BDPA was quenched by the DMSO/D_2_O solution (94.5% (v/v) DMSO‐*d*_6_/5.0% D_2_O/0.5%(v/v) dichloroacetic acid‐*d*_2_ (DCA‐*d*_2_), pH* 5.4) and the NMR spectra of the protein were measured in the DMSO/D_2_O solution (Chandak et al. 2013; Kuwajima et al. 2022). To analyze the H/D‐exchange kinetics of individually identified NH groups of ^15^ N‐labeled BDPA, we first made their spectral assignment in the ^1^H–^15^ N HSQC spectrum of the protein in the DMSO/H_2_O solution (94.5% (v/v) DMSO‐*d*_6_/5.0% (v/v) H_2_O/0.5% (v/v) DCA‐*d*_2_, pH 5.4). Figure 3 shows the ^1^H–^15^ N HSQC spectrum of the uniformly ^13^C/^15^ N‐labeled BDPA in the DMSO/H_2_O solution. Using the combination of three‐dimensional spectral measurements, we successfully assigned all the backbone NH peaks observed in the HSQC spectrum (see Section 4). Although the three‐dimensional structure of native BDPA in aqueous solution contains three helices, BDPA in DMSO exhibited no secondary structures as estimated by the torsion angle prediction based on the backbone chemical shifts.

### 2.3 The H/D‐exchange kinetics of B domain of protein A NH groups

**FIGURE 4:**
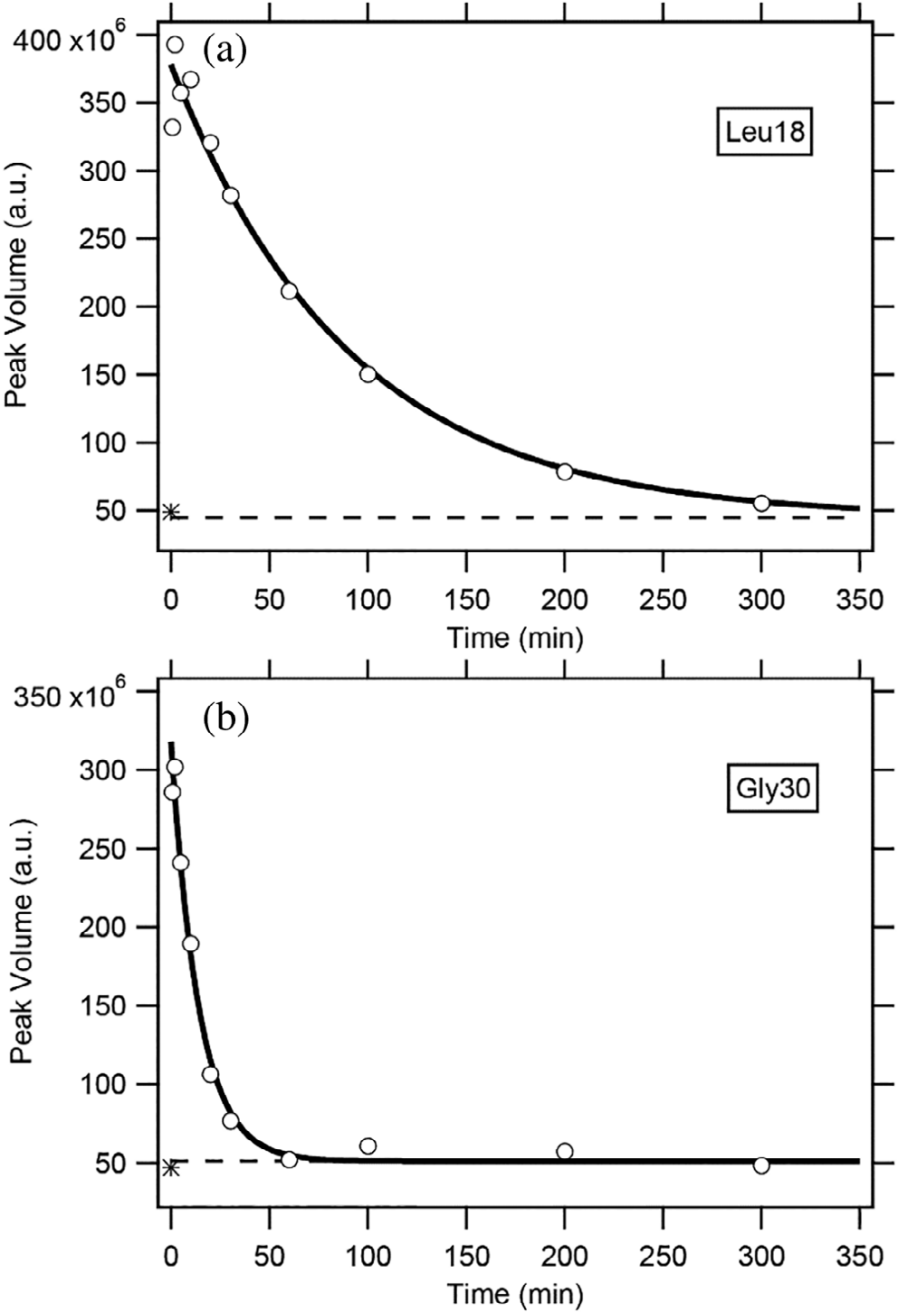
The H/D‐exchange curves for Leu18 (a) and Gly30 (b) in 6.0 M GdmCl at pH* 3.4 and 15.0°C. The solid lines are the theoretical curves best‐fitted to a single exponential function (Equation 7). A broken line in each panel indicates the theoretically estimated peak volume after complete exchange (i.e., *Y*(∞) in Equation 7), and an asterisk (*) in each panel, located at 50 × 10^6^ of the peak volume, indicates the experimentally observed value after heating the sample at 50°C for 30 min. Because the reaction mixtures contained 10% H_2_O, the final peak volumes did not reach zero. The *k*_obs_ values for the two residues are as follows: (a) (1.11 ± 0.17) × 10^−2^ min^−1^ and (b) (7.10 ± 0.73) × 10^−2^ min^−1^; the errors given are fitting error estimates.

The H/D‐exchange reactions of peptide NH protons of ^15^ N‐labeled BDPA were carried out in 6.0 M GdmCl at pH* 3.4 and 15.0°C. At pre‐determined exchange times, the H/D‐exchange reaction was quenched, and the H/D‐exchange buffer was replaced with the DMSO/D_2_O solution using a spin desalting column (see Section 4). The NMR spectra of the protein with the pre‐determined H/D‐exchange times were measured in the DMSO/D_2_O solution. Based on the backbone assignment of the ^1^H–^15^ N HSQC spectrum (Figure 3), we investigated the H/D‐exchange kinetics of all the individually identified NH protons. The exchange kinetics were given by the volumes of cross peaks in the HSQC spectra as a function of the exchange time. Excluding NH protons whose cross peaks could not be used as probes because of severe overlapping, we successfully followed the H/D‐exchange kinetics of 29 NH protons. All the exchange kinetics were well represented by a single‐exponential function (Equation 7). Figure 4 shows typical H/D‐exchange curves of the NH protons for two residues, Leu18 and Gly30. The values of the observed exchange rate constant (*k*_obs_) for the 29 NH protons are summarized in Table 2.

**TABLE 2:** The H/D‐exchange parameters of the backbone NH groups of unfolded BDPA at 6 M GdmCl (pH* 3.4 and 15°C) and the H‐bond lengths, acceptors, and secondary structures in the native structure (PDB code: 1BDD)

Residue	*k*_obs_ × 10^2^ (min^−1^)^a^	*k*_int_ × 10^2^ (min^−1^)	*P*	H‐bond length (Å)^b^	Acceptor residue^c^	Secondary structure^d^
A2	–	8.52	–			Coil
D3	–	27.88	–			Coil
N4	–	67.93	–			Coil
K5	5.15 ± 0.08	12.49	2.4			Coil
F6	–	4.99				Turn
N7	–	23.20				Turn
K8	–	12.49				Turn
E9	4.02 ± 0.17	14.36	3.6	2.4	N7	Turn
Q10	–	17.46				α‐Helix
Q11	–	11.92				α‐Helix
N12	–	32.02				α‐Helix
A13	4.72 ± 0.25	13.80	2.9	1.7	E9	α‐Helix
F14	2.61 ± 0.13	3.82	1.5	1.9	Q10	α‐Helix
Y15	2.01 ± 0.05	4.06	2.0	2.4	Q11	α‐Helix
E16	3.26 ± 0.08	12.22	3.7	1.8	A13	α‐Helix
I17	1.22 ± 0.05	2.84	2.3	2.0	A13	α‐Helix
L18	1.03 ± 0.07	1.03	1.0	2.4	F14	α‐Helix
H19	–	25.43		2.3	E16	Coil
L20	–	11.63				Turn
P21	n.a.	n.a.	n.a.			Turn
N22	8.32 ± 0.43^e^	25.40	3.1	2.6	L20	Turn
L23	–	3.63		2.5	L20	Turn
N24	5.18 ± 0.54	12.49	2.4			Coil
E25	–	22.76				α‐Helix
E26	–	25.29				α‐Helix
Q27	–	17.46				α‐Helix
R28	–	12.48		2.4	N24	α‐Helix
N29	13.36 ± 1.09	33.53	2.5	1.7	E25	α‐Helix
G30	8.11 ± 0.42	12.83	1.6	2.4	E26	α‐Helix
F31	–	5.65		2.4	R28	α‐Helix
I32	–	1.41		2.6	R28	α‐Helix
Q33	–	4.43		1.8	N29	α‐Helix
S34	7.42 ± 0.18	24.31	3.3	1.9	F31	α‐Helix
L35	2.58 ± 0.11	3.45	1.3	2.5	F31	α‐Helix
K36	–	3.72		2.1	I32	α‐Helix
D37	–	36.50		2.3	Q33	α‐Helix
D38	–	93.07		1.7	S34	Turn
P39	n.a.	n.a.	n.a.			Turn
S40	8.36 ± 1.00^e^	19.28	2.3			Turn
Q41	–	14.99				α‐Helix
S42	9.53 ± 0.45	24.31	2.6			α‐Helix
A43	6.15 ± 0.12	13.12	2.1			α‐Helix
N44	9.99 ± 0.82	20.24	2.0	2.4	Q41	α‐Helix
L45	1.86 ± 0.11	3.63	2.0	2.4	Q41	α‐Helix
L46	–	1.10		2.3	S42	α‐Helix
A47	1.94 ± 0.12	4.18	2.1	2.4	A43	α‐Helix
E48	3.84 ± 0.27	10.93	2.8	2.1	L45	α‐Helix
A49	2.93 ± 0.16	15.30	5.2	2.3	L45	α‐Helix
K50	–	6.01		2.3	L46	α‐Helix
K51	1.50 ± 0.15	7.88	5.2	2.4	A47	α‐Helix
L52	1.54 ± 0.08	2.29	1.5	2.1	E48	α‐Helix
N53	7.53 ± 0.31	12.49	1.7	1.9	A49	α‐Helix
D54	–	57.82		1.9	K50	α‐Helix
A55	5.50 ± 0.60	22.23	4.0	2.3	K51	α‐Helix
Q56	4.00 ± 0.12	7.57	1.9	2.5	L52	α‐Helix
A57	2.51 ± 0.13	10.46	4.2	1.9	N53	Coil
P58	n.a.	n.a.	n.a.			Coil
K59	2.74 ± 0.18^e^	3.63	1.3			Coil

^a^ The *k*_obs_ values were obtained by three independent H/D‐exchange measurements, and the average values and the standard errors are shown for all residues.

^b^ The H‐bond distance between the peptide amide H atom of the residue in the first column and the peptide carbonyl O atom of the acceptor residue in native BDPA. The residue pairs that have a distance less than or equal to 2.6 Å are listed as the H‐bonded pairs.

^c^ The peptide CO group of the acceptor residue making an H‐bond with the NH group of the residue in the first column. The H‐bond information was obtained by the VADAR web server (http://vadar.wishartlab.com/index.html) (Willard et al. 2003).

^d^ The secondary structures of native BDPA determined by the program STRIDE (http://webclu.bio.wzw.tum.de/cgi‐bin/stride/stridecgi.py) (Heinig and Frishman 2004).

^e^ The *k*_int_ values of these residues (N22, S40, and K59), following the proline residues, depend on the *cis–trans* isomer state of the proline residue (Bai et al. 1993), and the *k*_int_ is given by:
$$k_{\mathrm{int}}=0.2\cdot k_{\mathrm{int},\mathrm{cis}}+0.8\cdot k_{\mathrm{int},\text{trans}},$$
where *k*_int,cis_ and *k*_int,trans_ are the intrinsic exchange rate constants of the *cis* and *trans* isomers, respectively, and we have assumed that 80% of each proline residue is in the *trans* form in 6.0 M GdmCl.

The protection factor (*P*), which is the degree of protection of a given NH group against the H/D exchange with a solvent deuteron, is given by the ratio of the intrinsic rate constant (*k*_int_) of the chemical H/D‐exchange reaction to *k*_obs_ of the NH group as follows:

(1) $$P=\frac{k_{\mathrm{int}}}{k_{\mathrm{obs}}}$$

The *k*_int_ is sensitive to neighboring residues, solution conditions (pH and temperature), and equilibrium and kinetic isotope effects, and it is quantitatively estimated from the parameters for side‐chain effects of the neighboring residues and the reference H/D‐exchange rate constant (*k*_ref_) of poly‐DL‐alanine (PDLA), which is in a randomly coiled state in aqueous solution (Bai et al. 1993; Connelly et al. 1993). The *k*_ref_ values at different pD values in 0 M and 6.0 MGdmCl are given in Appendix S1. The *k*_int_ of each NH group of BDPA was calculated by Equation (9), and the corresponding *P* value was given by Equation (1). The *k*_ref_ values of PDLA at 0 M and 6.0 M GdmCl are coincident at pD 3.7 (i.e., pH* 3.3) (Figure S1), suggesting that the *k*_int_ values of BDPA evaluated here at 6.0 M GdmCl are very close to those at 0 M GdmCl. From the *P* values thus obtained, some of the above 29 NH groups were fully exposed to solvent without any significant protection against H/D exchange (*P* < 2); the 4 NH groups of Ala49, Lys51, Ala55, and Ala57 were significantly protected (*P* ≥ 4.0), and the additional 17 NH groups of Lys5, Glu9, Ala13, Tyr15, Glu16, Ile17, Asn22, Asn24, Asn29, Ser34, Ser40, Ser42, Ala43, Asn44, Leu45, Ala47, and Glu48 showed a *P* value in the range 2.0 ≤ *P* < 4.0. As a result, 21 out of the 29 NH protons exhibit a *P* value of 2.0–5.2, indicating that BDPA unfolded in 6.0 M GdmCl retains residual structures that protect the NH groups from the H/D‐exchange reactions. Nguyen et al. (2018) reported that an increase of the reference value (*B* in Equations 8 and 9) by a small factor of 1.35 brought the predicted *k*_int_ values into excellent agreement with the experimental data for unstructured apolipoprotein C3. If we introduce the same correction into the *k*_int_ values in Table 2, the *P* value will increase by a factor of 1.35.

## 3 DISCUSSION

Here, we have investigated the H/D‐exchange behavior of BDPA in 6 M GdmCl at pH* 3.4 and 15°C by DMSO‐quenched H/D‐exchange 2D NMR spectroscopy with the use of spin desalting columns for medium exchange. This is thus the first report to demonstrate the presence of residual structures in BDPA in a concentrated denaturant at an amino‐acid residue resolution. The folding behavior of BDPA has been studied intensively in both real experiments (Gouda et al. 1992, 1998; Kato et al. 1993; Deisenhofer 1981; Sato et al. 2004, 2006; Myers and Oas 2001; Arora et al. 2004; Huang et al. 2007, 2009; Bottomley et al. 1994; Bai et al. 1997; Cedergren et al. 1993; Dimitriadis et al. 2004; Vu et al. 2004; Davis et al. 2015; Otosu et al. 2017; Oikawa et al. 2013, 2015) and computational simulations (Garcia and Onuchic 2003; Lei et al. 2008; Shao and Gao 2011; Shao and Zhu 2017; Jani et al. 2016; Shao 2014; Skolnick et al. 1993; Kolinski and Skolnick 1994; Boczko and Brooks 3rd. 1995; Guo et al. 1997; Zhou and Karplus 1999; Alonso and Daggett 2000; Berriz and Shakhnovich 2001; Favrin et al. 2002; Ghosh et al. 2002; Islam et al. 2002; Zhou and Linhananta 2002; Kussell et al. 2002; Jang et al. 2003). Experimentally, the equilibria and kinetics of the BDPA folding/unfolding reactions were well approximated by a two‐state model, in which only the N and U states were significantly populated (Sato et al. 2004, 2006; Myers and Oas 2001; Arora et al. 2004; Huang et al. 2007, 2009; Bottomley et al. 1994; Bai et al. 1997; Cedergren et al. 1993; Dimitriadis et al. 2004), and the transition state present at the free‐energy barrier between N and U was characterized by a Φ‐value analysis (Sato et al. 2004, 2006). However, more recent studies, using laser‐induced temperature jumps coupled with time‐resolved infrared spectroscopy with site‐specific probes, have provided direct evidence for the presence of an early folding intermediate, in which the signals of partial formation of helices H1 and H3 were observed with an apparent time constant of ~100 ns (Vu et al. 2004; Davis et al. 2015). Analyses of peptide fragments corresponding to helices H1, H2, and H3 using CD and NMR spectra have also shown that the H3 helix fragment has the highest helical propensity, exhibiting a 25–30% helicity at pH 5 in aqueous solution (Bai et al. 1997; Haack et al. 1997). Recent all‐atom molecular dynamics (MDs) simulations, utilizing new methodologies such as replica exchange, integrated tempering sampling, and temperature‐aided cascade MD, have successfully simulated the full process of folding of BDPA from unfolded conformations to the N state, indicating that the most stable helix, H3, is formed first, followed by H2 and then H1 (Garcia and Onuchic 2003; Lei et al. 2008; Shao and Gao 2011; Shao and Zhu 2017; Jani et al. 2016; Shao 2014). Recently, the structural heterogeneity of unfolded BDPA in concentrated GdmCl has also been revealed by single‐molecule fluorescence resonance energy transfer time‐series measurements (Oikawa et al. 2013, 2015) and by 2D fluorescence lifetime correlation spectroscopy (Otosu et al. 2017), apparently implicating the presence of residual structures in unfolded BDPA; however, these studies did not provide structural information at the amino acid residue level. In the following, we further discuss: (1) Residual helical structures of BDPA in 6 M GdmCl; (2) Consistent interpretation of the CD and the H/D‐exchange results; and finally, (3) Possible folding mechanisms and folding initiation sites of BDPA, deduced from the present results.

### 3.1 Residual helical structures of B domain of protein A in 6 M GdmCl

From Table 2, 21 out of the 29 NH protons identified in the HSQC spectrum were significantly protected with a *P* value of 2.0–5.2, indicating the residual structures were retained in unfolded BDPA. Because the protein was unfolded in 6 M GdmCl, it is most likely that these NH protons were protected by formation of an H‐bond with a certain acceptor group. When the H/D‐exchange protection is brought about by formation of the H‐bond with a specific acceptor group with much faster fluctuations than the intrinsic exchange rate, we can estimate the fraction of H‐bonding (*f*_H‐bond_) for the protected NH groups on the basis of the EX2 mechanism of the H/D exchange. Because only the non‐H‐bonded form of the NH proton is available for H/D exchange, (1 – *f*_H‐bond_) is equal to *k*_obs_/*k*_int_ (= 1/*P*). Therefore, it follows that

(2) $$f_{\text{Hbond}}=1-\frac{1}{P}$$

When NH protons have *P* values larger than 2.0 and 5.2, the *f*_H‐bond_ values are larger than 0.50 and 0.81, respectively, from Equation (2). The free energy of the H‐bond breakage (0.0–0.82 kcal/mol) estimated from the *f*_H‐bond_ values is thus much smaller than the unfolding free energy Δ*G*°_NU_ (5.3 kcal/mol) (Table 1). Nevertheless, the *f*_H‐bond_ values larger than 0.5 should be significant when we consider the kinetic folding mechanisms of the protein, and some of these weakly protected residues may play an important role in the formation of folding initiation sites at an initial stage of kinetic folding of the protein from the U state (see below).

The majority of the protected residues are involved in the native α‐helical regions (Table 2), strongly suggesting that these residues may participate in formation of the secondary structure framework during the kinetic refolding of the protein (see below). However, the *P* values of the residues are all much less than 10, indicating that the residues are fluctuating between the H‐bonded (i.e., α‐helical) and the non‐bonded (disordered) structures. The present study has also revealed that the degree of protection of H/D‐exchange in BDPA in 6 M GdmCl differs among the three native helical regions (H1 [residues 10–19], H2 [25–38], and H3 [41–57]). Intriguingly, the most highly protected residues are located in the H3 region, while H1 and H2 were less protected (Table 2). The findings of Shao and Gao (2011) support this observation: in their MD unfolding simulations of BDPA in 4 M GdmCl at 300 K, helix H3 was the most stable, while the formation of helices H1 and H2 required the packing of the hydrophobic core between them (Shao and Gao 2011).

The NH proton of Glu9 is significantly protected with a *P* value of 3.6 (*f*_H‐bond_ = 0.72), and this NH group forms a weak H‐bond with the peptide CO group of Asn7 in the native structure. This region of the protein adopts a very flexible structure (Gouda et al. 1992), and the weak H‐bonding interactions in flexible regions might be partially retained in the U state. The NH proton of Asn22 is significantly protected, with a *P* value of 3.1 (*f*_H‐bond_ = 0.68). The NH groups of Asn22 and Leu23 form local H‐bonds with the peptide CO group of Leu20, and these residues form a type I β‐turn in the native structure (PDB: 1BDD) (Gouda et al. 1992). This β‐turn structure may thus be partially retained in 6 M GdmCl. Six residues (Lys5, Glu9, Asn24, Ser40, Ser42, and Ala43) are significantly protected, but they do not have their backbone H‐bonding acceptors in native BDPA (Table 2). The NH protons of these residues, except for Ala43, may thus be protected by non‐native H‐bonding interactions in 6 M GdmCl. The NH proton of Ala43 is H‐bonded with the side‐chain O^γ^ atom of Ser42 in the native structure (PDB: 1BDD) (Gouda et al. 1992), and hence the same local backbone to side chain H‐bond might be partially retained in 6 M GdmCl.

### 3.2 Consistent interpretation of the circular dichroism and the H/D‐exchange results

While the DMSO‐quenched H/D‐exchange NMR data indicate the presence of the H‐bonded structures in the H1–H3 regions, such a residual secondary structure was not detected in the CD spectrum. Although the *f*_H‐bond_ values of the residual helical structures of BDPA ranged from 0.5 to 0.8 (*P* = 2.0–5.2) (Table 2), the mean residue ellipticity at 222 nm, [*θ*]_222_, was close to zero in 6.0 M GdmCl (Figure 2), apparently suggesting that BDPA in 6 M GdmCl was in a fully unfolded, unordered state. This apparent discrepancy may be reasonably interpreted in terms of incipient fluctuating helices of the protein in 6 M GdmCl.

Because of the end effects of helices, the CD intensity of a short helix strongly depends on the helix length (Chen et al. 1974; Gans et al. 1991; Woody 1996), and the mean residue ellipticity at 222 nm, which is mainly caused by the nπ* transition of the peptide CO group, is represented by

(3) $${\left[\theta \right]}_{222,n}={\left[\theta \right]}_{222,\infty }\left(n-k\right)/n,$$

where [*θ*]_222,*n*_ is the mean residue ellipticity of a helix of *n* peptide groups, [*θ*]_222,∞_ is the mean residue ellipticity of an infinite helix, and *k* is an empirical constant (*k*/*n* = 1 when *k* > *n*). Gans et al. (1991) analyzed the results for the calculated nπ* rotational strength of the Pauling and Corey α‐helix and obtained *k* = 4.6. Woody (1996) reported that experimental [*θ*]_222_ values for a number of small peptides reported by Jackson et al. (1991) gave *k* = 4.3 and [*θ*]_222,∞_ = −41,000 deg cm^2^/dmol. Equation (3) and these *k* values thus indicate that a short α‐helix with only one turn (*n* = 4) exhibits zero ellipticity.

Chin et al. (2002) studied CD spectra of short, fixed‐nucleus alanine helices. They used a 12‐residue sequence (Ac‐DKDGDGYISAAE‐NH_2_) taken from an EF hand protein, calmodulin; the four C‐terminal peptide units in this sequence become helical when the peptide is bound to La^3+^ (Siedlecka et al. 1999). By adding alanine residues at the C‐terminus, they studied the CD spectra of helices with 4, 8, and 11 peptide units (i.e., one‐turn, two‐turn, and three‐turn helices). Their results show that the *k* value in Equation (3) is dependent on *n*: *k* = 2.8 for the one‐turn helix (*n* = 4), *k* = 3.5 for the two‐turn helix (*n* = 8), and *k* = 4.0 for the three‐turn helix (*n* = 11). If we apply *k* = 2.8 to Equation (3) for the one‐turn helix, the [*θ*]_222,4_ value becomes 0.3 × [*θ*]_222,∞_ = −12,300 deg cm^2^/dmol, much larger in intensity than the ellipticity observed experimentally (Figure 2). However, unlike the weak protections of NH protons of BDPA in 6 M GdmCl, the one‐turn helix of the La^3+^‐bound 12‐residue peptide is surprisingly stable and remains almost fully helical from 4 to 65°C. Furthermore, the peptide CD spectrum of the one‐turn helix reported by Chin et al. (2002) was constructed by subtraction of the spectrum of the La^3+^‐depleted apo‐peptide, which forms a disordered structure, from the spectrum of the La^3+^‐bound holo‐peptide, in which the C‐terminal four peptide units form the one‐turn helix. Therefore, effects of the La^3+^ binding on the CD spectrum of the non‐helical region of the peptide may not be eliminated by this subtraction; the La^3+^ is strongly coordinated with the two side‐chain carboxylates of Asp5 and Glu12 of the holo‐peptide (PDB code: 1NKF) (Siedlecka et al. 1999). We thus hypothesize that BDPA forms incipient, fluctuating and mostly one‐turn helices in 6 M GdmCl and that the CD intensity of such incipient fluctuating helices is close to zero at 222 nm, as observed in Figure 2. Hence, the DMSO‐quenched H/D‐exchange method provides a useful tool for the sensitive detection of residual structures that are unobservable in CD spectroscopy.

### 3.3 Possible folding mechanisms and folding initiation sites of B domain of protein A

From Table 2, we have the *P* values of the 21 NH protons that form an α‐helical H‐bond in the native structure, and 14 (67%) of these 21 NH protons show a *P* value larger than or equal to 2.0 (*f*_H‐bond_ ≥0.5) in 6 M GdmCl, strongly suggesting that these weakly protected NH protons may form much stronger α‐helical H‐bonds under native folding conditions. Interestingly, the recent studies by Dyer's group (Vu et al. 2004; Davis et al. 2015) have revealed the presence of an α‐helical folding intermediate formed with a time constant of ~100 ns by laser‐induced temperature jumps combined with time‐resolved infrared spectroscopy. Recent all‐atom MD folding simulations of BDPA have also demonstrated the presence of the early α‐helical intermediate (Garcia and Onuchic 2003; Lei et al. 2008; Shao and Gao 2011; Shao and Zhu 2017; Jani et al. 2016; Shao 2014). These recent studies are thus fully consistent with our observation that the NH protons involved in the native α‐helices are significantly protected even in the U state in 6 M GdmCl, and it is strongly suggested that these weakly protected NH protons lead to the α‐helical folding intermediate at an early stage of kinetic refolding in the native folding conditions.

Traditionally, stopped‐flow CD techniques (Kuwajima 1996; Arai and Kuwajima 2000; Woody 2004) and pulse‐labeling H/D‐exchange combined with 2D NMR or mass spectrometric analysis (Krishna et al. 2004; Englander et al. 2016) have been used for detecting and characterizing the α‐helical folding intermediate. However, BDPA is a very fast folding protein: its kinetic refolding reaction from the U state has a time constant less than 10 μs (Myers and Oas 2001; Dimitriadis et al. 2004). The folding of BDPA is thus too fast to detect a possible kinetic intermediate by these conventional techniques, and until recently, the folding reaction of this protein was treated as a simple two‐state process from U to N.

Among the three native helical regions, H1, H2, and H3, the H3 region has the highest average *P* value (Table 2), suggesting that helix H3 is also the most resistant against unfolding among the three helices. This is consistent with the previous helix‐fragment studies of BDPA, which has shown the highest helical propensity of the isolated H3 fragment (Bai et al. 1997; Haack et al. 1997), and also consistent with the MD unfolding simulations in concentrated GdmCl (Shao and Gao 2011) and at a high temperature (Alonso and Daggett 2000), both of which have shown that the H3 helix is most resistant against unfolding. The helical propensity calculated by the Agadir program (http://agadir.crg.es) (Muñoz and Serrano 1994) based on the helix/coil transition theory was also highest for H3.

**FIGURE 5:**
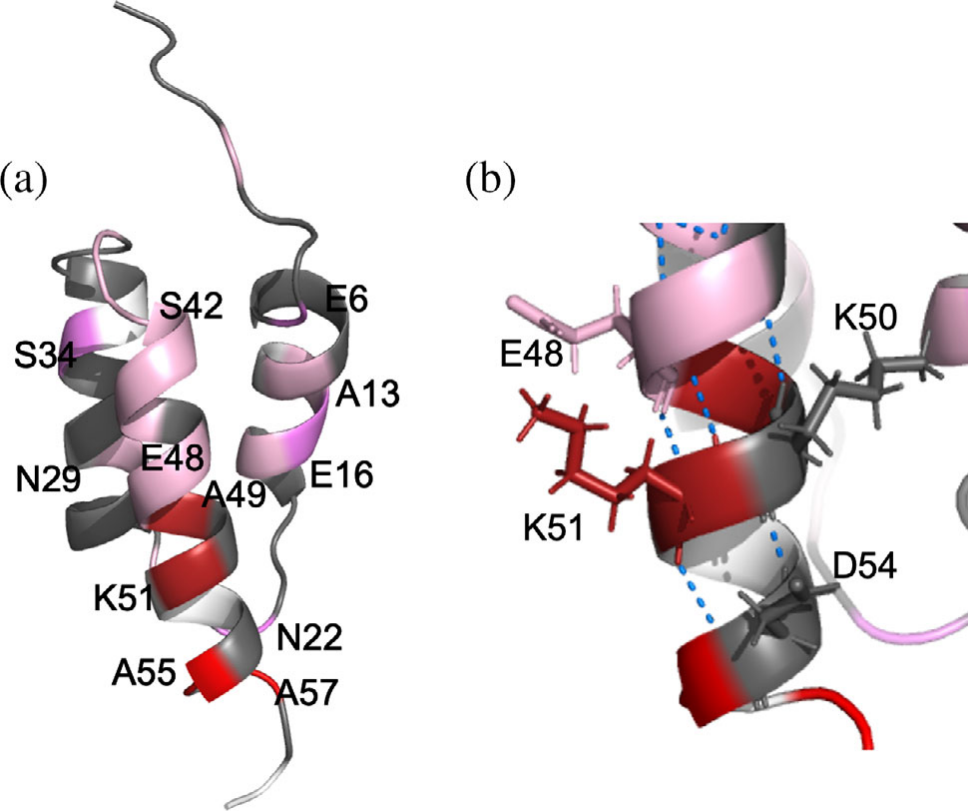
Mapping of the *P* value on the three‐dimensional structure of native B domain of protein A (BDPA). The *P* values of the residues are mapped on the structure of BDPA (PDBID: 1BDD). The residues with *P* values larger than 2.0, 3.0, 4.0, and 5.0 are colored in light magenta, magenta, red, and dark red, respectively. (a) The whole structure of BDPA. (b) A close‐up view of the H3 helix region. The H‐bonds are shown as blue dashed lines.

Among the NH groups involved in helix H3, those of Ala49 and Lys51 show the highest protection factor, *P =* 5.2 (*f*_H‐bond_ = 0.81), in 6 M GdmCl (Table 2). The formation of H‐bonded one‐turn helices of these residues brings the side chains of Glu48 and Lys51 into close proximity, promoting salt‐bridge formation between them (Figure 5). The Lys50–Asp54 pair may also form a salt bridge and stabilize the H3 helix, although the *P* value of Asp54 is unfortunately lacking in Table 2. In fact, it is well‐known that the (Glu^−^/Asp^−^)–Lys^+^ salt‐bridge (or the [Glu^0^/Asp^0^]–Lys^+^ singly charged H‐bond at acid pH) significantly stabilizes the helical structure in short peptides (Marqusee and Baldwin 1987; Chakrabartty and Baldwin 1995). The potential suppression of the electrostatic effect in the salt bridge formation in 6 M GdmCl suggests that the residual structures in the U state may be much more stable under native conditions with physiological salt concentrations. Therefore, we hypothesize that the middle portion of helix H3, residues 48–54, acts as a folding initiation site of BDPA, and leads to the α‐helical folding intermediate as described above. In our previous study on the H/D‐exchange of Ub (ubiquitin) by the DMSO‐quenched 2D NMR spectroscopy, we found similar salt‐bridge pairs between positions *i* and *i* + 3, that is, Glu24‐Lys27 and Lys29‐Asp32, in the middle α‐helix of the protein, and the NH groups of these residues showed significantly high *P* values in 6 M GdmCl (Yagi‐Utsumi et al. 2020). The present study together with the previous Ub study (Yagi‐Utsumi et al. 2020) thus underscore that the salt‐bridge formation between positively and negatively charged side‐chains embedded in segments with high helical propensity are important for the formation of residual structures, which can be propagated into the intermediate with the native‐like α‐helix under folding conditions.

Sato et al. (2004, 2006) analyzed the transition state for folding of BDPA by means of an experimental Φ‐value analysis. They found that the H3 helix was poorly formed in the transition state, while the H2 helix was well formed with many stabilizing tertiary hydrophobic interactions (Sato et al. 2004, 2006). They proposed that their results were inconsistent with a classical framework mechanism (Kim and Baldwin 1990), in which the native secondary structure is formed before the tertiary structure, but better represented by a nucleation‐condensation mechanism, in which the secondary and tertiary structures are formed in parallel in a cooperative manner (Daggett and Fersht 2003). Therefore, their results are inconsistent with the present results as well as the recent experimental (Vu et al. 2004; Davis et al. 2015) and computational studies (Garcia and Onuchic 2003; Lei et al. 2008; Shao and Gao 2011; Shao and Zhu 2017; Jani et al. 2016; Shao 2014), which clearly demonstrate that the BDPA folding from the U state accumulates the early α‐helical intermediate, and hence it follows the framework mechanism. The reason for this discrepancy remains to be determined, but their application of the two‐state model to the non‐two‐state folding of BDPA might have played a role.

DMSO‐quenched H/D‐exchange 2D NMR spectroscopy with the use of spin desalting columns (Chandak et al. 2013; Kuwajima et al. 2022) is thus very useful for detecting residual secondary structures in the U state and deducing a possible structured folding intermediate for proteins, especially when the proteins fold very rapidly with a time constant much less than ~1 ms, so that the intermediate cannot be detected by conventionally used techniques such as stopped‐flow or pulse‐labeling H/D‐exchange 2D NMR.

## 4 MATERIALS AND METHODS

### 4.1 Materials

DMSO‐*d*_6_ and D_2_O were purchased from Cambridge Isotope Laboratories (Tewksbury, MA). DCA‐*d*_2_ and PDLA were purchased from Sigma–Aldrich (St. Louis, MO). DCA‐*d*_2_ was used as a pH buffer agent of the quenching DMSO/D_2_O solution (94.5% DMSO‐*d*_6_/0.5% DCA‐*d*_2_/5% D_2_O [pH* 5.4]). GdmCl was purchased from Nacalai Tesque (Kyoto, Japan). Deuterated GdmCl was produced by repeated cycles of dissolution of GdmCl in D_2_O followed by lyophilization. ^15^ N‐labeled and ^13^C/^15^ N‐double‐labeled BDPA for hetero‐nuclear NMR measurements was expressed in *Escherichia coli* host cells BL21(DE3), which were purchased from SMOBIO Technology Inc. (Hsinchu, Taiwan), at 37°C in M9 minimal medium. Non‐labeled BDPA was expressed in LB broth as a recombinant protein and purified as described previously (Yagi‐Utsumi et al. 2020). The concentration of BDPA was determined spectrophotometrically in a microvolume spectrophotometer (NanoDrop One) using molar extinction coefficients of 1490 M^−1^ cm^−1^ at 0 M GdmCl and 1285 M^−1^ cm^−1^ at 6.0 M GdmCl at 280 nm (Pace et al. 1995).

### 4.2 Circular dichroism measurements

CD measurements were carried out at 15.0°C in a Jasco model J‐1500 CD spectropolarimeter with a Peltier thermostatted cell holder. The spectra of BDPA were measured in the peptide region below 250 nm at a protein concentration of 61 μM, and the pathlength of the optical cuvette was 0.10 cm. The spectra were represented by mean residue ellipticity [*θ*], in units of deg·cm^2^/dmol.

### 4.3 NMR measurements

To achieve the backbone resonance assignment of ^13^C/^15^ N‐labeled BDPA (1 mM) in the DMSO/H_2_O solution (94.5% [v/v] DMSO‐*d*_6_/5% [v/v] H_2_O/0.5% [v/v] DCA‐*d*_2_), three‐dimensional HN(CA)NNH, HNCA, HN(CO)CA, HNCO, HNCACO CBCA(CO)NH, and HNCACB experiments were performed at 25°C on a Bruker AVANCE‐800 spectrometer with a cryogenic probe. The ^1^H–^15^ N HSQC spectra were recorded at a ^1^H observation frequency of 800 MHz with 256 (*t*_1_) × 2048 (*t*_2_) complex points and eight scans per *t*_1_ increment.

The standard ^1^H–^15^ N HSQC spectra of ^15^ N‐labeled BDPA in the DMSO/D_2_O solution with different H/D‐exchange times were acquired at 25°C. The spectra were recorded at a ^1^H observation frequency of 800 MHz with 98 (*t*_1_) × 1024 (*t*_2_) complex points and 16 scans per *t*_1_ increment. All NMR data were processed using TopSpin (Bruker, Billerica, MA) and Sparky (Lee et al. 2015). Secondary structural elements were identified based on the backbone chemical shifts by using the program TALOS (Shen et al. 2009) (https://spin.niddk.nih.gov/NMRPipe/talos/).

### 4.4 Dimethylsulfoxide‐quenched H/D‐exchange experiments of B domain of protein A

The H/D‐exchange reaction of unfolded BDPA was started by 10‐fold dilutions of 3 mM ^15^ N‐labeled BDPA unfolded in 6.0 M GdmCl–0.10 M NaCl in H_2_O (pH 3.6) into 6.0 M deuterated GdmCl–0.10 M NaCl–11.1 mM sodium formate in D_2_O at pH* 3.2 and 15.0°C. The pH* value of the reaction mixture was 3.4 at room temperature, and the pD value was 3.8 (= pH* + 0.4). At each pre‐determined exchange time, 1.0 mL of the reaction mixture was taken, the reaction was quenched in liquid nitrogen, and the frozen mixture was kept at −80°C until the medium exchange and the subsequent NMR measurement. For the NMR measurement, the frozen sample was thawed by immersing the sample in a water bath previously set at 7°C, and the medium containing 6.0 M GdmCl was exchanged for the quenching DMSO/D_2_O solution by centrifugation using a spin desalting column (Zeba Spin Desalting Column 89891; Thermo Fisher Scientific K.K., Tokyo) at 20°C. The use of spin desalting columns for the medium exchange in the DMSO‐quenched H/D‐exchange experiments is superior to conventionally used refolding and lyophilization. The details of the use of spin desalting columns were reported previously (Chandak et al. 2013; Kuwajima et al. 2022; Yagi‐Utsumi et al. 2020).

### 4.5 H/D‐exchange experiments of poly‐DL‐alanine

To obtain the reference exchange rate constant *k*_ref_, we carried out the H/D‐exchange experiments of PDLA at different pD values between 1.5 and 4.5 in the absence and presence of 6.0 M GdmCl at 15.0°C. The H/D‐exchange reaction was started by 10‐fold dilution of 2–3 mg/mL PDLA in 0 M (or 6.0 M) GdmCl–0.10 M NaCl in H_2_O into 0 M (or 6.0 M deuterated GdmCl)–0.10 M NaCl–11.1 mM buffer salt in D_2_O at 15.0°C. The buffer salts used were sodium dihydrogen‐phosphate (pH* < 2.5), sodium dihydrogen‐citrate (2.5 < pH* < 3.6) and sodium hydrogen‐succinate (pH* > 3.6). The H/D‐exchange reactions were directly measured by one‐dimensional (1D) NMR spectroscopy at ^1^H frequencies of 400 MHz and 500 MHz on a Bruker AVANCE‐400 spectrometer (experiments at 6.0 M GdmCl) and a Bruker AVANCE‐500 spectrometer (experiments at 0 M GdmCl), the latter of which was equipped with a cryogenic probe. The H/D‐exchange data of PDLA are given in Appendix S1.

### 4.6 Equilibrium unfolding of B domain of protein A

The equilibrium unfolding of BDPA induced by GdmCl was observed by measuring the CD ellipticity at 222 nm (Figure 2b). The unfolding was well approximated with a reversible two‐state transition between the N and the U states as

(4) $$N\overset{K_{\mathrm{NU}}}{⇄}U,$$

where *K*_NU_ is the equilibrium constant. The standard Gibbs free‐energy change of unfolding, Δ*G*_NU_, is proportional to the molar concentration of GdmCl, [GdmCl], and is given by:

(5) $$\Delta G_{\mathrm{NU}}=-\mathrm{RT}\ln K_{\mathrm{NU}}=\Delta G_{\mathrm{NU}}^{\circ }-m_{\mathrm{NU}}\left[\text{GdmCl}\right],$$

where *R* and *T* are the gas constant and the absolute temperature, respectively, Δ*G*°_NU_ is the Δ*G*_NU_ at [GdmCl] = 0, and *m*_NU_ is the dependence of Δ*G*_NU_ on [GdmCl]. The equilibrium unfolding transition curve measured by the average residue molar ellipticity (*θ*_obs_) as a function of [GdmCl] was fitted to the following equation by the method of nonlinear least‐squares, from which we evaluated Δ*G*°_NU_ and *m*_NU_ (Santoro and Bolen 1992):

(6) $$\theta_{\mathrm{obs}}=\frac{\left(k_{0}+k_{1}\text{[GdmCl]}\right)+\left(k_{2}+k_{3}\text{[GdmCl]}\right)\exp\left(\frac{-\Delta G_{\mathrm{NU}}^{\circ }+m_{\mathrm{NU}}\text{[GdmCl]}}{\mathrm{RT}}\right)}{1+\exp\left(\frac{-\Delta G_{\mathrm{NU}}^{\circ }+m_{\mathrm{NU}}\text{[GdmCl]}}{\mathrm{RT}}\right)},$$

where the terms (*k*_0_ + *k*_1_[GdmCl]) and (*k*_2_ + *k*_3_[GdmCl]) represent the *θ*_obs_ values of the protein in the pure N and the pure U states, respectively. The nonlinear least‐squares curve fitting of the unfolding data was performed by using the IGOR Pro 8 software package (WaveMetrix, San Diego, CA).

### 4.7 Analysis of the H/D‐exchange kinetics

The observed H/D‐exchange curves of BDPA and PDLA, given by the NMR signal intensity *Y*(*t*) as a function of the H/D‐exchange time *t*, were single‐exponential under the present conditions, and fitted to the following equation:

(7) $$Y\left(t\right)=\Delta Y\cdot e^{-k_{\mathrm{obs}}t}+Y\left(\infty \right),$$

where Δ*Y*, *k*_obs_, and *Y*(∞) are the kinetic amplitude, the observed H/D‐exchange rate constant, and the final value of the signal intensity, respectively. The *Y*(*t*) values are given by cross‐peak volumes in the 2D ^1^H–^15^ N HSQC spectra of BDPA, and by peak areas in the 1D NMR spectra of PDLA.

The reference exchange rate constant, *k*_ref_, of PDLA was measured as a function of pD under the present condition (6.0 M GdmCl–0.10 M NaCl–10 mM buffer salt, and 15.0°C) by 1D NMR spectroscopy. The log*k*_ref_ shows a V‐shaped dependence on pD, which is determined by the two second‐order rate constants for specific acid catalysis (*A*) and specific base catalysis (*B*) and the first‐order rate constant for water catalysis (*C*) (Figure S1). The *k*_ref_ of PDLA is thus given by

(8) $$\begin{array}{l} k_{\mathrm{ref}}=A\cdot \left[D^{+}\right]+B\cdot \left[{\mathrm{OD}}^{-}\right]+C\\ \;=A\cdot 10^{-\mathrm{pD}}+B\cdot 10^{\left(\mathrm{pD}-pKw\right)}+C \end{array}$$

where *K*_w_ is the ionization constant of D_2_O, and the p*K*_w_ value of D_2_O is 15.24 at 15.0°C (Covington et al. 1966). From the pD dependence of the *k*_ref_, we obtained *A*, *B*, and *C* (see Appendix S1). The *k*_int_ of each NH group of BDPA includes side‐chain effects of the amino acid residues adjacent to the NH group, and it is represented by the following equation:

(9) $$k_{\mathrm{int}}=A_{L}\cdot A_{R}\cdot A\cdot \left[D^{+}\right]+B_{L}\cdot B_{R}\cdot B\cdot \left[{\mathrm{OD}}^{-}\right]+B_{L}\cdot B_{R}\cdot C,$$

where *A*_L_, *A*_R_, *B*_L_, and *B*_R_ represent the side‐chain effects, and subscripts L and R refer to peptide groups to the left and right of the side chain, respectively. The values of *A*_L_, *A*_R_, *B*_L_, and *B*_R_ were given by Bai et al. (1993) and slightly improved by Nguyen et al. (2018).

## AUTHOR CONTRIBUTIONS

**Saeko Yanaka:** Conceptualization (equal); data curation (equal); formal analysis (equal); investigation (equal); writing – original draft (equal); writing – review and editing (equal). **Maho Yagi‐Utsumi:** Conceptualization (equal); data curation (equal); formal analysis (equal); investigation (equal); writing – review and editing (equal). **Koichi Kato:** Conceptualization (equal). **Kunihiro Kuwajima:** Conceptualization (equal); data curation (equal); formal analysis (equal); funding acquisition (equal); investigation (equal); supervision (equal); writing – original draft (equal); writing – review and editing (equal).

## Supporting information


**Appendix S1:** Supporting Information

## Data Availability

Assigned chemical shift data for BDPA in the DMSO/H2O solution (94.5% (v/v) DMSO‐d6/5% (v/v) H2O/0.5% (v/v) DCA‐d2) were deposited in the BMRB under the accession number 51541.
