# Supplementary material for: The B domain of protein A retains residual structures in 6 M guanidinium chloride as revealed by hydrogen/deuterium‐exchange NMR spectroscopy
Source: Protein Sci. 2023 Feb 14;32(3):e4569. doi: 10.1002/pro.4569 (PMC9926473; doi:10.1002/pro.4569)
Supplement: Supplementary file 1 — Appendix S1: Supporting Information [file PRO-32-e4569-s001.pdf]

The B Domain of Protein A Retains Residual Structures in 6 M Guanidinium Chloride as Revealed by Hydrogen/Deuterium-Exchange NMR Spectroscopy

Saeko Yanaka[1,2,3] | Maho Yagi-Utsumi[1,2,3] | Koichi Kato[1,2,3] | Kunihiro Kuwajima[4]

[1]Exploratory Research Center on Life and Living Systems (ExCELLS) and Institute for Molecular Science (IMS), National Institutes of Natural Sciences, 5-1 Higashiyama, Myodaiji, Okazaki, Aichi 444-8787, Japan
[2]Department of Functional Molecular Science, School of Physical Sciences, SOKENDAI (the Graduate University for Advanced Studies), 5-1 Higashiyama, Myodaiji, Okazaki, Aichi 444-8787, Japan
[3]Graduate School of Pharmaceutical Sciences, Nagoya City University, 3-1 Tanabe-dori, Mizuho-ku, Nagoya, Aichi 467-8603, Japan
[4]Department of Physics, School of Science, the University of Tokyo, 7-3-1 Hongo, Bunkyo-ku, Tokyo 113-0033, Japan

Correspondence
Koichi Kato, Exploratory Research Center on Life and Living Systems (ExCELLS) and Institute for Molecular Science (IMS), National Institutes of Natural Sciences, 5-1 Higashiyama, Myodaiji, Okazaki, Aichi 444-8787, Japan
Email: kkatonmr@ims.ac.jp
Kunihiro Kuwajima, Department of Physics, School of Science, the University of Tokyo, 7-3-1 Hongo, Bunkyo-ku, Tokyo 113-0033, Japan
Email: kuwajima@ims.ac.jp

Present Address
Saeko Yanaka, Graduate School of Pharmaceutical Sciences, Kyushu University, Fukuoka, 3-1-1 Maidashi, Higashi-ku, Fukuoka, Fukuoka, 812-8582, Japan

SUPPORTING INFORMATION

Alanine reference exchange rate

To obtain the intrinsic chemical exchange rate constant k_{int} of each NH group of BDPA, we measured the reference exchange rate constants k_{ref} of PDLA at 0 M GdmCl and at 6.0 M GdmCl as a function of pD at 15.0°C. PDLA is a model for a random peptide, and its NH H/D-exchange rate constant is taken as k_{ref} to estimate the k_{int} values of the NH groups of globular proteins[1]. The effect of GdmCl on the k_{ref} of PDLA was reported by Loftus *et al.*[2], but the k_{ref} values in the base catalyzed region (pH* > 3.5) at 0 M GdmCl reported in their study were 2.5 times smaller than those reported by Bai *et al.*[1] Therefore, we here reexamined the k_{ref} values at 0 M and 6.0 M GdmCl. The results are shown in Fig. S1. The pD dependence of k_{ref} is given by Eq. (8), and the best fit values of the logarithmic catalytic constants (log A, log B and log C) are shown in Table S1. The values obtained at 0 M GdmCl are in reasonable agreement with those reported by Bai *et al.*[1] (Table S1). The log A, log B and log C values at 6.0 M GdmCl were used for calculating the k_{int} value of each NH group of BDPA.

Table S1. H/D-exchange rate constants for PDLA at 15.0°C

GdmCl concentration (M)	log A (M^{-1} min^{-1})	log B (M^{-1} min^{-1})	log C (min^{-1})	Reference
0	1.64	10.07	−1.93	This study
6.0	1.11	10.22	−2.33	This study
0[a]	1.44	10.02	−1.75	Bai *et al.* (1993)[1]

[a]The values were calculated from those at 20°C reported by Bai *et al.* (1993)[1] using activation energies of the H/D-exchange reactions and the difference in pK_w between 15°C and 20°C.

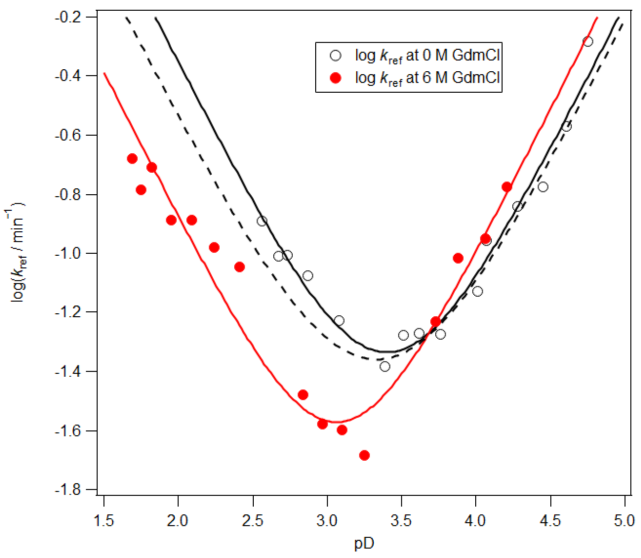

FIGURE S1 The dependence of k_{ref} of PDLA on pD at 0 M GdmCl (open circles) and at 6.0 M GdmCl (red filled circles) in the presence of 0.10 M NaCl at 15.0°C. The black and red solid lines represent the theoretically best fit to the following equation:

$$\log k_{\mathrm{ref}} = \log \{A \cdot 10^{-\mathrm{pD}} + B \cdot 10^{(\mathrm{pD} - \mathrm{p}K_{\mathrm{w}})} + C\},$$

using the parameter values shown in Table S1. A broken line indicates the theoretical curve based on the parameter values reported by Bai *et al.*[1] with correction for temperature difference.

REFERENCES

1. Bai Y, Milne JS, Mayne L, Englander SW. Primary structure effects on peptide group hydrogen exchange. Proteins. 1993;17:75-86.
2. Loftus D, Gbenle GO, Kim PS, Baldwin RL. Effects of denaturants on amide proton-exchange rates: A test for structure in protein-fragments and folding intermediates. Biochemistry. 1986;25:1428-1436.
